# High-mobility group box 1 protein, receptor for advanced glycation end products and nucleosomes increases after marathon

**DOI:** 10.3389/fphys.2023.1118127

**Published:** 2023-02-14

**Authors:** Julia Schoenfeld, Astrid Roeh, Stefan Holdenrieder, Pia von Korn, Bernhard Haller, Kimberly Krueger, Peter Falkai, Martin Halle, Alkomiet Hasan, Johannes Scherr

**Affiliations:** ^1^ Department of Prevention and Sports Medicine, University Hospital Klinikum rechts der Isar, Technical University of Munich, Munich, Germany; ^2^ DZHK (German Center for Cardiovascular Research), Partner Site Munich Heart Alliance, Munich, Germany; ^3^ Department of Psychiatry, Psychotherapy and Psychosomatics, Medical Faculty, University of Augsburg, Bezirkskrankenhaus Augsburg, Augsburg, Germany; ^4^ Department of Psychiatry and Psychotherapy, University Hospital, Ludwig-Maximilians University Munich, Munich, Germany; ^5^ Institute of Laboratory Medicine, German Heart Centre Munich, Technical University Munich, Munich, Germany; ^6^ Institute of Medical Informatics, Statistics and Epidemiology, Klinikum rechts der Isar, Technical University of Munich, Munich, Germany; ^7^ University Center for Preventive and Sports Medicine, Balgrist University Hospital, University of Zurich, Zurich, Switzerland

**Keywords:** biomarker, necrosis, healthy, HMGB1, sRAGE, exercise

## Abstract

**Background:** Prolonged and strenuous exercise has been linked to potential exercise-induced myocardial damages. One potential key to unmask the discussed underlying mechanisms of this subclinical cardiac damage could be markers of immunogenic cell damage (ICD). We investigated the kinetics of high-mobility group box 1 protein (HMGB1), soluble receptor for advanced glycation end products (sRAGE), nucleosomes, high sensitive troponin T (hs-TnT) and high sensitive C-reactive protein (hs-CRP) before and up to 12 weeks post-race and described associations with routine laboratory markers and physiological covariates.

**Methods:** In our prospective longitudinal study, 51 adults (82% males; 43 ± 9 years) were included. All participants underwent a cardiopulmonary evaluation 10–12 weeks pre-race. HMGB1, sRAGE, nucleosomes, hs-TnT and, hs-CRP were analysed 10–12 weeks prior, 1–2 weeks before, immediately, 24 h, 72 h, and 12 weeks post-race.

**Results:** HMGB1, sRAGE, nucleosomes and hs-TnT increased significantly from pre- to immediately post-race (0.82–2.79 ng/mL; 1132–1388 pg/mL; 9.24–56.65 ng/mL; 6–27 ng/L; *p* < 0.001) and returned to baseline within 24–72 h. Hs-CRP increased significantly 24 h post-race (0.88–11.5 mg/L; *p* < 0.001). Change in sRAGE was positively associated with change in hs-TnT (rs = 0.352, *p* = 0.011). Longer marathon finishing time was significantly associated with decreased levels of sRAGE [−9.2 pg/mL (*β* = −9.2, SE = 2.2, *p* < 0.001)].

**Conclusion:** Prolonged and strenuous exercise increases markers of ICD immediately post-race, followed by a decrease within 72 h. An acute marathon event results in transient alterations of ICD, we assume that this is not solely driven by myocyte damages.

## 1 Introduction

The beneficial effects of regular moderate exercise on the cardiovascular system are well established. Regular exercise improves oxidative capacity (Irving et al., 2015), microvascular collateral formation (Möbius-Winkler et al., 2016) and contractile myocardial function (Nystoriak and Bhatnagar, 2018). Among endurance athletes, there is evidence that prolonged and strenuous exercise is associated with oxidative stress, increased cardiac volume load (Małek Ł et al., 2019) and oxidative DNA damage (Borghini et al., 2015) and may exert exercise-induced myocardial damage and even injury in terms of reversible fatigue or irreversible heart failure (Douglas et al., 1990). Furthermore, an increased risk of exercise-related sudden cardiac death (SCD) has been reported among endurance runners with pre-existing cardiac abnormalities, most likely caused by prolonged strenuous exercise (Albert et al., 2000).

This assumption is supported by studies from the 1990s which reported increased markers of cardiac and muscular damage like troponins (Tn) and creatine kinase (CK) in otherwise healthy individuals after heavy exercise and extreme endurance events such as the Berlin Marathon (Artner-Dworzak et al., 1990; Koller et al., 1995). Additionally, systematic reviews and meta-analyses within the last decade (Regwan et al., 2010; Sedaghat-Hamedani et al., 2015) showed increased Tn values above the 99th percentile (14 ng/L) in 83% of endurance athletes immediately after prolonged and strenuous exercise (Sedaghat-Hamedani et al., 2015). However, our group was able to demonstrate a return of Tn levels to baseline levels within 72 h after a marathon race (Scherr et al., 2011). The time of appearance of the absolute peak value and downslope differ from e.g. myocardial infarction with much faster kinetics pointing to a revesible effect, which leads to the conclusion that these are benign phenomenon. However, recent imaging studies suggest a compromised cardiomyocyte integrity (Aengevaeren et al., 2020).

One potential key to unmask the discussed underlying mechanisms of this subclinical cardiac damage could be markers of immunogenic cell damage (ICD) such as circulating nucleosomes, high-mobility group box 1 protein (HMGB1), and soluble receptor for advanced glycation end-products (sRAGE). Nucleosomes are basic components of nuclear chromatin and are formed by a complex of core histone proteins and DNA that is twisted around them. Linker DNA connects them to a nucleosomal chain (Koyama and Kurumizaka, 2017). After cleavage by specific endonucleases, they are released by dying and stressed cells into the blood circulation. HMGB1 is a nuclear non-histone-DNA-binding protein that stabilizes the nucleosome structure, enables the bending of DNA and facilitates transcription (Balliano et al., 2017). Upon cell stress, HMGB1 can be released in association with nucleosomes or dissociated from them (Bell et al., 2006). RAGE is an important cellular receptor and binding partner for HMGB1, S100 and other danger associated molecular pattern (DAMP) markers transmitting proinflammatory signals on the surface of antigen presenting cells, dendritic cells and macrophages (Pilzweger and Holdenrieder, 2015). However, RAGE can also be shed from these cells and act as a soluble anti-inflammatory decoy receptor by catching HMGB1 and other DAMPs (Bell et al., 2006). These immunogenic markers are released by stressed/damaged cells like muscle, immune, endothelial or liver cells by necrotic, apoptotic or NETosis pathways and therefore, are associated with cardiac cell stress, acute and chronic diseases like diabetes (Volz et al., 2010), arteriosclerosis (Andrassy et al., 2012), cancer (Stoetzer et al., 2012), trauma (Stahl et al., 2016), sepsis (Matsumoto et al., 2015), myocardial infarction (MI) (Sorensen et al., 2011), and heart failure (Volz et al., 2010; Pilzweger and Holdenrieder, 2015).

A recent study conducted by Beko and colleagues including 70 non-professional half marathon and marathon runners reported increased levels of HMGB1 and sRAGE immediately after the race. Both, HMGB1 and sRAGE returned to baseline values after two to 7 days of recovery (Bekos et al., 2016). Beko et al. were not able to detect an increase in sRAGE after a marathon race and they related this to the different training habits. However, they did not further evaluate the association with performance. Consistent with these observations of HMGB1 and sRAGE, circulating cell-free DNA (cfDNA), paralleled that of plasma HMGB1, increased in half marathon runners immediately after the race and decreased to baseline values within 2 h post-race (Atamaniuk et al., 2004). Although, the studies showed mostly an increase of these markers following exercise, many questions remain. To date, no study has evaluated the predictive factors e.g., performance, and laboratory markers associated with the increases of ICD and illuminated the sources of ICD. Therefore, we aimed to evaluate the kinetics of HMGB1, sRAGE, nucleosomes, hs-TnT and hs-CRP before, immediately after, and up to 12 weeks post-race after a marathon race. Furthermore, we identified routine markers of clinical chemistry (e.g., Creatinine and CK) and physiological covariates (exercise capacity, age, BMI and marathon finishing time) associated with post-race concentration of these biomarkers.

## 2 Method and design

### 2.1 Study participants

We included 51 marathon runners (of the initial 100 participants) of the longitudinal observational ReCaP-Study [Running effects on cognition and plasticity; details published previously (Roeh et al., 2019)] in our analyses. Participants were aged between 18 to 60 years, successfully registered for the Munich Marathon 2017, completed at least one half-marathon prior to the event, had sufficient German language skills and provided written informed consent. Participants with relevant neurological, cardiac or psychiatric diseases, pregnancy, cannabis abuse, and BMI >30 were excluded.

### 2.2 Ethical approval

The study has been approved by two local ethic committees: Ludwig-Maximilians University Munich (reference number 17-148) and the University Hospital Klinikum rechts der Isar, Technical University of Munich (reference number 218/17S). The trial was registered at DRKS-German Clinical Trials Register (DRKS-ID: DRKS00012496).

### 2.3 Study design

A total of six visits were performed. The first (baseline) visit took place 10–12 weeks (V-1) and the second visit 1–2 weeks (V0) prior to the marathon. The third, fourth, fifth, and sixth were immediately (V1), 24 h (V2.1), 72 h (V2.2) and 12 weeks (V3) after the marathon.

### 2.4 Examinations

All participants underwent a standardized physical examination and a full cardiac checkup at study site by trained medical staff. Body height and weight were measured *via* seca scale (Seca, 764, seca GmbH, Hamburg, Germany). Blood pressure (mmHg) was measured after resting in supine position for 5 min. Body fat was measured *via* calipometry and calculated according to the 7-folder formula of Jackson and Pollock (1978). A standardized 12-lead resting electrocardiography (ECG) was recorded (1 min duration, speed of 50 mm·s^−1^ and a voltage scale equivalent of 10 mm·mV^−1^) using Custo cardio 200 (custo diagnostics 3.8; custo med GmbH, Ottobrunn, Germany) in supine position. Additionally, physical fitness and chronotropic competence of all participants were measured *via* cardiopulmonary exercise test (CPET) including a 12-lead ECG on a treadmill by ramp protocol until voluntary exhaustion. Blood pressure was measured before the test and at maximum load, as well as one and 3 min after the test. Furthermore, a transthoracic echocardiography (IE33, Philips, Amsterdam, Netherlands; standard 2D parasternal short- and long-axis images and apical 2-, 3-, and 4-chamber views) was performed.

### 2.5 Blood sampling

At all six visits, blood samples were taken from the antecubital vein using two 10 mL lithium-heparin plasma tube, a 5 mL K2-EDTA plasma tube and a 10 mL gel serum tube (Sarstedt, Nuermbrecht, Germany). Samples were transported (<20 min) to the certified central laboratory of the German Heart Centre Munich where they were centrifuged and analyzed for routine markers of clinical chemistry such as creatinine and creatine kinase (CK) according to national quality standards. Residual samples of heparin, EDTA-plasma and serum were subsequently aliquoted into 2 mL cryotubes and stored at −80°C until further analyses.

#### 2.5.1 High-mobility group box 1 protein (HMGB1) and receptor for advanced glycation end products (Soluble RAGE)

HMGB1 and sRAGE concentration was assessed from serum tube by a sandwich enzyme linked immunosorbent assay (ELISA) according to the instructions of the manufacturer (HMGB1 ELISA, ST51011, TECAN IBL International GmbH, Hamburg, Germany; Quantikine^®^ Human RAGE ELISA, R&D Diagnostics, Minneapolis, MN) that was applied on a DS2 automated ELISA processing system (Dynex Technologies, Chantilly, VA, United States). The quantification of the results was done by use of a calibration curve with a measuring range of 0.625–80 ng/mL for HMGB1 and 78–5,000 pg/mL for RAGE.

#### 2.5.2 Nucleosomes

Nucleosomes were measured from serum tube by the Cell Death Detection ELISA^PLUS^ according to the instructions of the manufacturer (Cat. No. 11774425001, Version 15, Roche Diagnostics, Mannheim, Germany). The quantification of the nucleosomes results was done by use of a calibration curve with a measuring range of 2.83–241.5 ng/mL.

Methods for HMGB1, sRAGE, and nucleosomes were research-use-only (RUO) ELISA assays that were thoroughly validated for their analytical performance and for preanalytically influencing factors before use (Holdenrieder et al., 2001; Lehner et al., 2012; Wittwer et al., 2012). Measurements were done as single determinations. Standard curves and quality controls were included in every run. Serial samples of individuals were run in the same assays. Inter-assay variabilities of the different plates were checked by artificial and serum control materials. Thereby coefficients of variations (CVs) of HMGB1 were between 7.5% and 11.7%, of sRAGE between 5.4% and 8.1% and for nucleosomes at 6.4%.

#### 2.5.3 High sensitive Troponin T (hs-TnT)

Hs-TnT was measured from serum tube quantitatively by the highly sensitive electro-chemiluminescence-immunoassay (ECLIA) technology on a Cobas E411 analyser platform (Roche Diagnostics Deutschland GmbH, Mannheim, Germany). The limit of detection (LoD) of the method was 5 ng/L and the limit of quantification (LoQ) 13 ng/L. The reference limit of Hs-TnT in healthy volunteers (99th percentile) was 14 ng/L (Saenger et al., 2011).

#### 2.5.4 High sensitive C-reactive protein (hs-CRP)

Hs-CRP was measured from serum tube by a latex-particle amplified turbidimetry immunoassay method using a Cobas c 501 analyzer (Roche Diagnostics Deutschland GmbH, Mannheim, Germany) with a measurement range of 0.3–200.0 mg/L. The reference limit for adults was <3.0 mg/L (Ridker, 2003).

Methods for hs-cTnT and hs-CRP were IVD-CE labelled methods used in routine diagnostics of the Institute of Laboratory Medicine of the German Heart Centre Munich according the quality control system defined by the guidelines of the German Federal Medical Council (Rili-BÄK).

### 2.6 Statistical analysis

The statistical analysis was performed with IBM SPSS Statistics for Windows version 25 (IBM Corp., Armonk, NY, United States). The Gaussian distribution was tested using the Kolmogorov–Smirnov test. Normally distributed variables are presented as mean ± standard deviation (SD), non-normally distributed data as median and interquartile range (IQR), categorical variables as absolute (n) and relative (%) frequencies. Distributions of immunogenic markers at different visits were compared using Friedman’s test and between two visits by Wilcoxon signed-rank test; for categorical variables Cochran’s Q-test. Correlations between the variables were assessed using Pearson’s correlation or Spearman’s correlation coefficient. We analyzed the influence of the covariates physical performance, age, BMI and marathon finishing time on the increase of the biomarker with a linear regression model. All tests were performed two-sided with a significance level of *α* = 0.05.

## 3 Results

Out of the initial 100 participants, 51 were included in the present analysis. Reasons for exclusion from final analysis (exclusion reasons during the study period) were: time constraints (*n* = 18), internal sicknesses (*n* = 6), orthopedic disease (*n* = 7), intermediate visit missing for time reasons (*n* = 10), marathon not finished (*n* = 4), termination of the study due to personal reasons (*n* = 1), exclusion criterion for marathon at V-1 (*n* = 1) and missing blood samples (*n* = 2). Baseline characteristics of the study population [*n* = 51, aged 43 ± 9 years (82% male)] are shown in Table 1. A total of 27 (52%) of the included participants had a positive family history of cardiovascular diseases.

**TABLE 1:** Baseline characteristics of the study population.

Age [years]	43 ± 9
Sex Male n [%]	42 [82.4]
Weight [kg]	75.2 ± 12.6
Height [cm]	178.2 ± 9.1
BMI [kg/m^2^]	23.5 ± 2.5
Body fat [%]	16.6 ± 5.5
Female	22.2 ± 5.3
Male	15.4 ± 4.8
SBP [mmHg]	122.8 ± 11.3
DBP [mmHg]	80.7 ± 6.4
Comorbid disease	
Hypertension n [%]	2 [3.9]
Diabetes mellitus n [%]	—
Smoker n [%]	1 [2]
Ex-Smoker n [%]	10 [19.6]
Dyslipidemia n [%]	—
Obesity (BMI>25 kg/m^2^) n [%]	8 [16]
Family history of CVD n [%]	27 [52.9]
Performance, running time, and training distance	
RER_max_	1.1 ± 0.7
HR_max_ [1*min^−1^]	173.7 ± 16.3
Lactate_max_ [mmol* L^−1^]	7.5 ± 2.2
VO_2peak_ [ml*kg^−1^*min^−1^]	46.9 ± 6.5
Female	41.7 ± 7.7
Male	48.0 ± 5.7
Marathon time [min]	237.1 ± 36.7
Female	264.6 ± 48.0
Male	231.1 ± 31.4
Weekly training history [km* week^−1^]	42.3 ± 21.6
Annual training history [km* year^−1^]	1,862.8 ± 917.9

Note: *n* = 51; Data are expressed in mean ± standard deviation (SD) and numbers (n) and percentage [%].

BMI, body mass index; SBP, systolic blood pressure; DBP, diastolic blood pressure; V0_2peak_, oxygen consumption at peak exercise; RER, respiratory quotient; HR, heart rate; max, maximum; km, kilometer; min, minute.

### 3.1 Kinetics of the biomarkers

**FIGURE 1:**
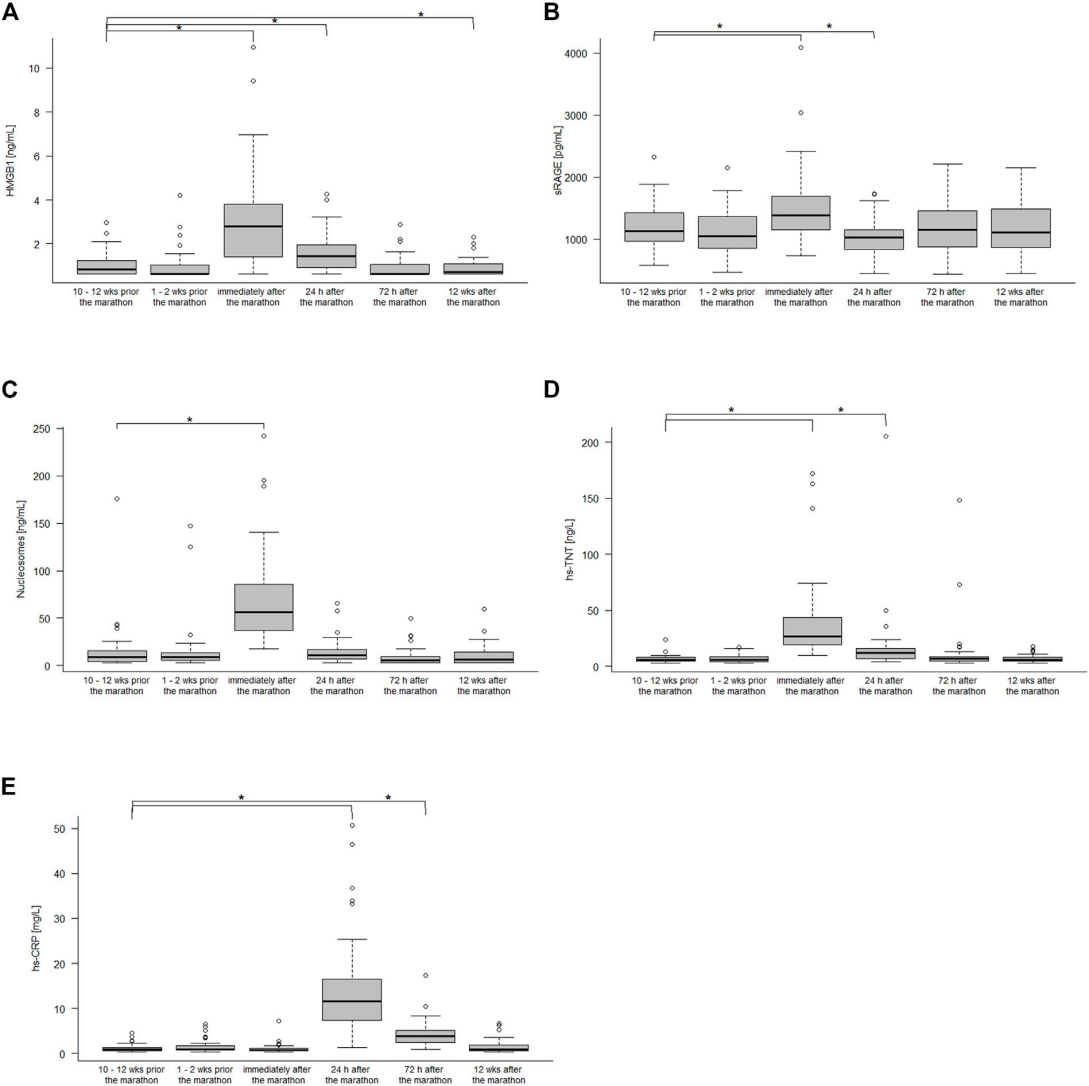
HMGB1 **(A)**, sRAGE **(B)**, nucleoseome **(C)**, hs-TnT **(D)**, and hs-CPR **(E)** concentration 10–12 weeks, 1–2 weeks before the marathon, immediately after the marathon, 24 h, 72 h after the marathon and 12 weeks after the marathon. Boxplots show median and the interquartile range (IQR); *p < 0.05 compared to the baseline values.

Figure 1 shows the kinetics of HMGB1, sRAGE, nucleosomes, hs-TnT and hs-CRP over the different measurement points. Comparison of the data at baseline to immediately after the marathon showed significant increases in sRAGE with medians 1,132 pg/mL (IQR 960–1,430 pg/mL) and 1,388 pg/mL (1,148–1,686 pg/mL, *p* < 0.001), in HMGB1 with medians of 0.82 ng/mL (IQR 0.62–1.27 ng/mL) and 2.79 ng/mL (1.37–3.87 ng/mL, *p* < 0.001), in nucleosomes with medians of 9.24 ng/mL (IQR 4.34–15.96 ng/mL) and 56.65 ng/mL (35.12–87.5 ng/mL, *p* < 0.001), and hs-TnT with medians of 6 ng/L (IQR 5–8 ng/L) and 27 ng/L (19–45 ng/L, *p* < 0.001). Hs-CRP increased with a peak at 24 h after the marathon (medians 0.88 mg/L (IQR 0.64–1.35 mg/L) and 11.5 mg/L (7.21–16.70 mg/L, *p* < 0.001) and remained elevated until 72 h 3.8 mg/L (2.34–5.13 mg/L, *p* < 0.001) after the marathon (Table2). Most remarkably, some runners had very high levels (>100 ng/L) of hs-TnT and/or some also very high levels of sRAGE, HMGB1 and nucleosomes 24 h post-race. In total, HMGB1 and hs-TnT were still slightly elevated after 24 h and returned to baseline 72 h after the marathon while sRAGE and nucleosomes reached baseline levels already after 24 h post-race.

**TABLE 2:** HMGB1, sRAGE, nucleoseomes, hs-TnT, and hs-CPR concentrations over the study period.

Markers	10–12 weeks prior the marathon	1–2 weeks prior the marathon	Immediately after the marathon	24 h after the marathon	72 h after the marathon	12 weeks after the marathon	
							*p*-value
sRAGE [pg/mL]	**1,132 (959–1,429)**	**1,048 (847–1,372)**	**1,388 (1,148–1,686)**	**1,021 (816–1153)**	**1,148(870–1,466)**	**1,104 (858–1,492)**	<0.001
		Δ −48.03 [-121.40, 13.56]	Δ 314.38 [166.33, 451.90]	Δ −153.64 [−232.04, −83.81]	Δ 8.41 [60.35, 77.17]	Δ −32.93 [-109.60, 29.77]	<0.001
HMGB1 [ng/mL]	**0.82 (0.62–1.27)**	**0.62 (0.62–1.06)**	**2.79 (1.37–3.87)**	**1.43 (0.86–2.00)**	**0.62 (0.62–1.10]**	**0.70 (0.62–1.09)**	<0.001
		Δ −0.04 [-0.18, 0.08]	Δ 2.02 [1.44, 2.60]	Δ 0.55 [0.28, 0.81]	Δ −0.08 [-0.17, 0.01]	Δ −0.12 [-0.23, −0.01]	<0.001
Nucleosomes [ng/mL]	**9.24 (4.34–15.96)**	**8.96 (5.22–13.65)**	**56.65 (35.12–87.5)**	**10.79 (6.66–17.62)**	**5.73 (2.83–10.00)**	**6.58 (2.83–14.17)**	<0.001
		Δ 0.34 [-3.67, 4.37]	Δ 54,04 [39.73, 68.35]	Δ −0.14 [-7.90, 7.61]	Δ −5.98[-13.14, 1.16]	Δ −4.25 [-11.89, 3.38]	<0.001
Hs-TnT [ng/L]	**6 (5–8)**	**6 (4–9)**	**27 (19–45)**	**12 (7–16)**	**7 (5–9)**	**6 (5–8)**	<0.001
		Δ 0.58 [-0.51, 1.71]	Δ 31.78 [21.96, 41.59]	Δ 10.06 [2.77, 17.34]	Δ 4.70 [-0.87, 10.27]	Δ −0.15 [-1.18, 0.86]	<0.001
Hs-CRP [mg/L]	**0.88 (0.64–1.35)**	**0.86 (0.71–1.72)**	**0.74 (0.54–1.10)**	**11.50 (7.21–16.70)**	**3.82 (2.34–5.13)**	**0.89 (0.62–1.98)**	<0.001
		Δ 0.34 [0.02, 0.67]	Δ −0.08 [-0.26, 0.10]	Δ 12.74 [10.08, 15.75]	Δ 3.10 [2.45, 3.75]	Δ 0.34 [0.03, 0.67]	<0.001
Cut off values*							
Hs-TnT >14 ng/L, n [%]	1 [2]	5 [10]	43 [90]	16 [31]	5 [10]	2 [4]	<0.001
Hs-CRP >3.0 mg/L, n [%]	2 [4]	4 [8]	1 [2]	49 [96]	33 [64]	4 [8]	<0.001

Note: *n* = 51 marathon runners; wks, weeks. Delta values are the difference between the prior value and the baseline value. Kinetics of cell death markers are presented as median and interquartile range (IQR), shown in bold. *p*-value over the measurement times were analysed using Friedman’s test; Alteration as difference mean (Δ) to 10–12 weeks prior the marathon (V-1) and 95% confidence interval [95% CI]; Cut off values are presented in numbers (n) and percentage [%]. *p*-value were analysed using Cochran’s Q-test.

HMGB1, sRAGE, nucleosomes, hs-TnT, and hs-CPR concentrations over the study period are summarized in Table 2.

One of the runners had a pre‐race (V-1) and 43 runners a post-race (V1) hs-TnT level above the upper reference limit of 14 ng/L (*p* < 0.001). In one runner (♂, 58 years, BMI 25.5 kg/m^2^, blood pressure 130/80 mmHg), hs-TnT was elevated at baseline, increased immediately and remained elevated until 72 h after the marathon (Baseline: 24 ng/L; immediately after the marathon: 58 ng/L; 24 h after the marathon: 205 ng/L; 72 h after the marathon: 148 ng/L; 12 weeks after the marathon: 7 ng/L). He revealed neither further cardiovascular risk factors nor symptoms in the course of the study, both initial examinations with cardiopulmonary exercise testing and subsequent examinations were unremarkable.

Overall, none of the runners reported symptoms suggestive of acute myocardial infarction during and/or after the race. Following a comprehensive clinical evaluation at baseline of all included individuals, there was no evidence for any clinically relevant cardiac abnormalities and pathologies.

### 3.2 Correlations of the biomarkers

Regarding the acute effects of marathon running, the following correlations were found: sRAGE immediately after the marathon correlated negatively with the finishing time (r = −0.541; *p* < 0.001; *n* = 48, Supplementary Figure S1). Changes in hs-TnT and hs-CRP or CK did not correlate with an increase in HMGB1 or nucleosomes (all *p* > 0.05). Only, the increase in sRAGE correlated positively with changes in hs-TnT (rs = 0.352, *p* = 0.011, *n* = 51), HMGB1 correlated positive with an increase in nucleosomes (r = 0.377, *p* = 0.006, *n* = 48), and a hs-TnT increase correlated significantly with an increase in hs-CRP (rs = 0.322, *p* = 0.021, *n* = 51).

### 3.3 Effects of covariates on the biomarkers

Linear regression models including age, BMI, marathon finishing time and VO_2peak_ (*R* ^2^ = 0.271, F (4, 44) = 5.45, *p* < 0.001) showed that every 1-min increase in marathon finishing time was associated with a sRAGE decrease at visit 1 of −9.2 pg/mL (regression coefficient b = −9.2, standard error = 2.2, *p* < 0.001), other variables were not significantly associated (BMI: b = −3.0 se = 27.2, *p* = 0.911; VO_2peak_: b = −19.72, se = 12.1, *p* = 0.110; age: b = 1.32, se = 6.6, *p* = 0.843).

## 4 Discussion

In this prospective observational study, we observed that sRAGE, HMGB1, nucleosomes and hs-TnT increased significantly directly after the marathon. Only hs-CRP increased- as expected- with a delay of 24 h after the marathon. Our findings concerning the kinetics of those markers after marathon complement and extend the work by Beiter et al. (2011); Scherr et al. (2011); Bekos et al. (2016). The three studies showed an increase of HMGB1, nucleosomes or TnT immediately after exhausting exercise in marathon or treadmill runners. Contrary to our results, Bekos et al. did not demonstrate an increase of RAGE immediately after a marathon (Bekos et al., 2016), but only after a half-marathon The author hypothesized that the missing increase of RAGE immediately after the marathon could be the result of training habits or exercise intensity. In this regard, we tested the hypothesis, that training had an influence on the expression of ICD markers, in our analysis. The fact that we found an association between RAGE and marathon finishing time—slower runners had a higher decrease in the RAGE units—suggests that training conditions influence expression of RAGE markers after a marathon. We could not demonstrate further associations between the expression of RAGE and performance parameters. When comparing studies described in the literature regarding training influences on RAGE expression, it shows inhomogeneous results, with studies indicating an inducible decrease of RAGE after training interventions (Kotani et al., 2011; Drosatos et al., 2021). Nevertheless, two studies indicated an increase of RAGE values within training interventions in cardiovascular risk patients (Choi et al., 2012; Sponder et al., 2018). In one randomized controlled trial with type 2 diabetes patients, RAGE levels increased after a 12-week aerobic exercise intervention (60 min at moderate intensity, 5 times/week) (Choi et al., 2012). In a second prospective study, the influence of long-term physical activity (8 months) on serum sRAGE levels in 98 participants was investigated. RAGE levels increased up to 22% in participants with a long-term performance gain of >5% compared to <4.9% (Sponder et al., 2018). Authors of the mentioned studies hypothesized that this variating pattern of decrease and increase in RAGE levels depending on exercise intensity, points towards the capacity of sRAGE to act as a marker for increased or decreased AGEs production (Choi et al., 2012; Sponder et al., 2018). In the present study, we could not demonstrate any association between HMGB1 or Nucleosomes and training intensity. However, previous studies reported that regular training could decrease HMGB1 levels (Gmiat et al., 2017) and that HMGB1 reacts depending on the intensity of the load. This can rather be attributed to an anti-inflammatory process and thus to the chronic long-term effects regarding training. In this case, HMGB1 and nucleosomes are low and sRAGE is rather elevated (as described above). This should be investigated in further studies.

Baseline values such as age, sex or body compositions may contribute to changes in biomarkers released into the circulation. Contrary to the literature, in which more studies indicate an inducible increase in RAGE and nucleosomes in older participants (Pinti et al., 2014; Recabarren-Leiva et al., 2021), we did not find an association between the kinetics of ICD markers and age or sex in our cohort. Only body fat correlated negatively with the increase in post-race sRAGE levels, consistent with existing literature (Dozio et al., 2017; Miranda et al., 2018). A possible explanation for this could be the homogeneous age level (43 ± 9 years) of the participants included in this study. Furthermore, it should be considered that our sample size consists only of 9 females and 42 males, which limits the statistical validity.

It is still unclear whether marathon running can lead to an irreversible cardiac damage or just a reversible cardiac fatigue. Comparing the current results with previous studies assessing the kinetics of TnT and ICM markers in patients, the patterns are significantly different (Apple et al., 1998). In patients after myocardial infarction, TnT and ICM kinetics are characterized by a steep peak increase and followed by prolonged elevation for at least 4–7 days (Apple et al., 1998; Kohno et al., 2009). Furthermore, we found a positive correlation between the increase in sRAGE and hs-TnT (rs = 0.352, *p* = 0.011), which is a different pattern to that reported in a previous study in patients with non-ST-segment elevation myocardial infarction. This observational study indicated that low levels of sRAGE are associated with high serum levels of cTnI and therefore, suggested that the severity of cardiac damage varies inversely with the levels of sRAGE (McNair et al., 2011). Finally, we plotted the increase in ICD markers from pre to post marathon in relation to the established cardiovascular risk factors (hypertension). We found a significant difference of 0.7 ± 1.0 vs. 2.0 ± 2.0, *p* = 0.031 in the change of HGMB1 in patients with hypertension compared to without hypertension. However, it should be noted that only two subjects were diagnosed with hypertension.

Since we could not detect any clear cardiac damage, the increases in the markers tend to originate from cells that play a greater role in terms of quantity (muscle cells, endothelia, leukocytes, CRP from liver cells, etc.) and less from perishing cardiac cells. The TnT increase can be explained by a release from muscle cells and the cytosol of the cardiomyocytes (10% of the TnT) (Bleier et al., 1998). The structurally bound fraction, however, is only released after the cardiomyocytes have perished, which can explain the days-long increases in TnT levels after an MI (Pilzweger and Holdenrieder, 2015). Regarding the ICM markers, a distinction must be made with regard to the reaction of the markers to acute and chronic effects. The acute effects only occur immediately after the marathon and are quickly compensated in a good training situation. In the acute phase, all three markers, nucleosomes, HMGB1 and sRAGE, are elevated. After the impact is absent again, the markers will decrease. At least for HMGB1 it is known that–after cessation of the damaging stimulus–it may contribute to regeneration of the tissue and preservation of the organ function on a local level. Therefore, HMGB1 after an acute marathon is hypothesized to be released from inflammatory cells and exerts protective functions for the heart preventing pathological remodeling (Germani et al., 2007). Recent studies suggest that the release of nucleosomes attributes either a rapid, active release mechanism from immune- or endothelial cells, or a passive shear stress-induced detachment mechanism from the cell surface area (Neuberger and Simon, 2022). The typical cell death mechanisms such as necrosis, apoptosis, and “suicidal NETosis (Brinkmann et al., 2004)”, in which chromatin is released from dying neutrophils after chromatin de-condensation and subsequent rupture of the nuclear and cell membrane, requires hours to complete (Neuberger and Simon, 2022). In contrast, the “vital” NETosis, in which detachment occurs passively from the cell surface can happen very rapidly (Pfister, 2022). As described in Pfister, in the vital NETosis, vesicles, containing nuclear DNA from neutrophils, merge with plasma membrane eventually resulting in an alive anuclear cytoplast (Pfister, 2022). Therefore, it seems to be well imaginable that “vital” NETosis is a major mechanism for the release of nucleosomes after marathon. A prolonged increase of nucleosomes following marathon however could originate either from reduced elimination and/or subsequent cell death originating from damaged tissues. In conclusion, the transient increase in these parameters may not reflect a cardiovascular strain. Further studies should investigate the mentioned other originating cell forms.

### 4.1 Limitations

There are some limitations that should be considered. The sample size of 51 marathon runners is relatively small. However, we investigated our cohort in the context of the training as well as in the recovery period over a after over a 6-month time period. In addition, the recovery period was determined to be 12 weeks after the marathon, which makes it unlikely to detect chronic pathological findings, especially as severe events in a young and healthy study population are rare. Furthermore, cut off values could be addressed only for hs-TnT and CRP. Values for sRAGE, HMGB1 and nucleosome still need to be explored. Future studies should therefore investigate the individual differences over time more closely and examine the cut off values for sRAGE and nucleosomes in order to support clinicians interpreting potential pathological alterations. To evaluate the pathomechanisms of the investigated biomarkers, additional clinical routine markers were taken in account. Nevertheless, specific and unspecific cardiac and pro inflammation pathways with more allocation to a specific organ tend to be more insightful. For example, inclusion of additional biomarkers such as CK-MB, interleukins, myeloperoxidase (MPO), active neutrophil elastase (NE), and proteinase 3 (PR3) should be considered.

## 5 Conclusion

In our study, markers of immunogenic cellular damage increased significantly in the early period after prolonged and strenuous exercise and returned to baseline values after a recovery phase of 24–72 h. Based on our results, it seems as if a marathon event does not lead to necrotic alterations but rather points to transient ischemic alterations instead. Therefore, there seems to be no increased risk for persistent cardiac necrosis if no underlying pathology is present. Furthermore, the training condition of active patients should be observed, as more intensive training or a regular training rhythm can influence the expression of the evaluated biomarkers.

## Data Availability

The raw data supporting the conclusion of this article will be made available by the authors, without undue reservation.
