# Supplementary figures and images for: High-mobility group box 1 protein, receptor for advanced glycation end products and nucleosomes increases after marathon
Source: Front Physiol. 2023 Feb 14;14:1118127. doi: 10.3389/fphys.2023.1118127 (PMC9971726; doi:10.3389/fphys.2023.1118127)

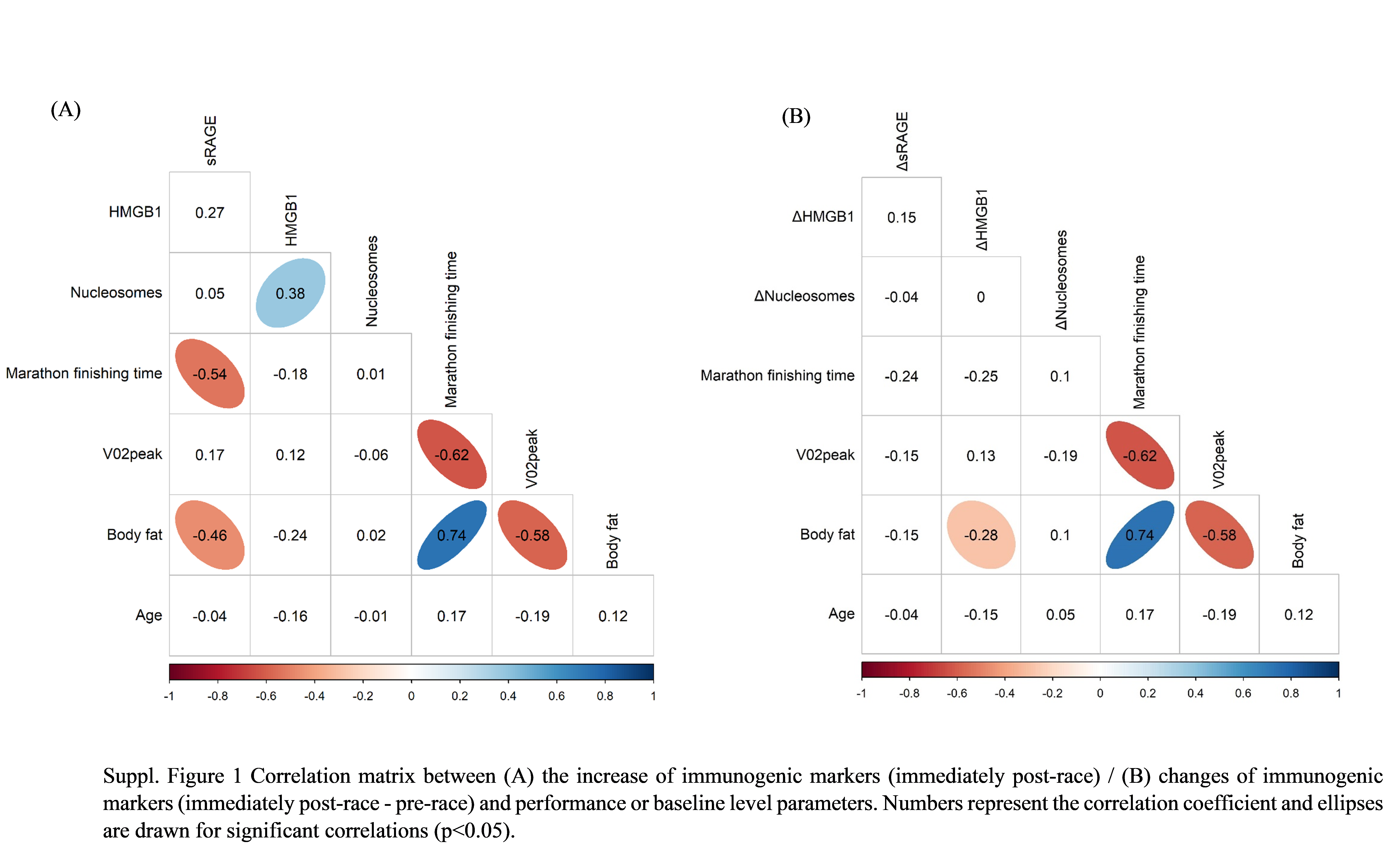

Supplement: Supplementary file 1 [file Image1.TIF]
